# A Low-Cost Approach for Batch Separation, Identification and Quantification of Microplastics in Agriculture Soil

**DOI:** 10.3390/toxics11050461

**Published:** 2023-05-16

**Authors:** Shan Zhang, Wenfeng Li, Anming Bao, Gongxu Jia, Xiaoli Lin, Qingling Zhang

**Affiliations:** 1 State Key Laboratory of Desert and Oasis Ecology, Xinjiang Institute of Ecology and Geography, Chinese Academy of Sciences, Urumqi 830011, China; 2 Key Laboratory of GIS & RS Application Xinjiang Uygur Autonomous Region, Urumqi 830011, China; 3 University of Chinese Academy of Sciences, Beijing 100049, China; 4 Xinjiang Key Laboratory of Environmental Pollution and Bioremediation, Xinjiang Institute of Ecology and Geography, Chinese Academy of Sciences, Urumqi 830011, China; 5 Research Center for Ecology and Environment for Central Asia, Chinese Academy of Sciences, Urumqi 830011, China; 6 Shenzhen Key Laboratory of Intelligent Microsatellite Constellation, Shenzhen Campus of Sun Yat-sen University, Shenzhen 518107, China; 7 School of Aeronautics and Astronautics, Sun Yat-sen University, Shenzhen 518107, China

**Keywords:** a batch approach, microplastics separation, microplastics identification model, less time and low-cost, agriculture soil

## Abstract

An increasing trend of research on microplastics (MPs) pollution in soil requires plenty of accurate data on MPs occurrence in soil samples. Efficient and economical methods of obtaining MP data are in development, especially for film MPs. We focused on MPs originating from agricultural mulching films (AMF) and presented an approach that can separate MPs in batches and identify them quickly. It mainly includes separation by ultrasonic cleaning and centrifugation, digestion of organic matter, and an AMF-MPs identification model. Adding olive oil or n-hexane to saturated sodium chloride constituted the best combination of separation solutions. Controlled experiments proved that the optimized methods improved the efficiency of this approach. The AMF-MPs identification model provides specific characteristics of MPs and can identify MPs efficiently. Evaluation results showed that the mean MP recovery rate reached 95%. The practical application demonstrated that this approach could conduct MPs analysis in soil samples in batches with less time and low cost.

## 1. Introduction

With rapid social and economic development worldwide, plastic has become an indispensable product in modern society and has been widely used [1]. Millions of metric tons of plastics are used annually for various purposes [2]. As a direct consequence, plastic residuals are increasingly accumulating in natural environments [3]. Microplastic (MP), often defined as plastic particles less than 5 mm in length [4], can affect agroecosystem functioning by accumulating heavy metals or organic contaminants [5,6]. Some studies show that microplastics are widely distributed around the world, whether in the ocean [7], on land [8], on islands with little human activity [9], or even in polar glaciers [10]. Currently, microplastic, as a class of emerging and near-permanent contaminants, has received growing attention worldwide [11,12]. The current research shows that human activities and agricultural production dump a large number of plastics into soils, which can potentially impact soil ecosystems, crops, and livestock [13]. Moreover, agricultural mulching film (AMF) has been confirmed to be an important source of MP contamination in terrestrial environments [3,14]. In order to investigate the contamination in detail, we need a large amount of data on microplastic particles in soils.

The most commonly used and effective method for separating MPs from soil is density separation [15,16], which is based on the density difference between microplastics and environmental matrices, and a floatation procedure can be used for separation. However, such a procedure can easily cause some of the MPs with adhered impurities to be lost. Some methods use oil-water mixtures as a solution to separate MPs by freezing samples or settling the mixture solution in a separatory funnel [17,18,19], which are based on oil separation and require low temperatures. Other separation methods include froth flotation [20,21,22], magnetic extraction [23,24], electrostatic separation [25,26], and solvent extraction separation [27,28]. All these methods mentioned above can only be performed on a small number of samples at a time.

Moreover, current microplastic identification methods mainly include visual inspection, Raman spectroscopy, and Fourier-transform infrared (FTIR) spectroscopy. Visual inspection obtains the surface texture and other characteristics of obvious/possible MPs with a microscope and then directly identifies MPs [29,30,31]. This method is low-cost and convenient, but it is also time-consuming because there are no specific standards for the identification characteristics. Raman spectroscopy and FTIR spectroscopy are the two most commonly used methods, which are based on vibrational spectroscopy [32,33,34]. Both of them can achieve high identification accuracy for identifying MPs. In order to accurately reflect the actual situation of MP pollution in soils, it is often necessary to process and analyze a large number of soil samples. However, existing methods for microplastic extraction and identification have limited efficiency and high experimental cost, hindering the analysis of large batches of soil samples.

The purpose of our research is to develop a low-cost and efficient approach for batch-separating MPs from soil samples and identifying MPs accurately and quickly. This approach combined density separation and oil separation to increase the recovery rate for MPs. Inspired by Grause et al. [35], we adopted centrifugation to decrease the separation time.

Olive oil has a stronger affinity with polymers than other oils [17] and is thus chosen for microplastic separation. This research focused on MPs that stem from agricultural mulching film (AMF). The material in AMF is polyethylene (PE), which is mainly classified into three types: low-density polyethylene (LDPE), linear low-density polyethylene (LLDPE), and high-density polyethylene (HDPE).

## 2. Materials and Methods

### 2.1. Sample Collection and Pretreatment

Soil samples in this study were collected from plastic-mulched cotton fields in the Bayingol Mongolian Autonomous Prefecture (35°38′ N, 93°51′ E to 43°34′ N, 82°28′ E) (Figure S1), which is an arid region in Xinjiang, China. Samples were randomly collected from the 0–25 cm layer of topsoil (A horizon) with a soil auger and then stored in glass jars or paper bags. 

After being taken back indoors, the soil samples were put into aluminum foil containers, briefly rolled to loosen them, and then air-dried in a clean and airtight room for 3 to 5 days. Meanwhile, large pieces of plastics, straws, stones, and other impurities in the soil samples were removed with a 5 mm mesh sieve. Some microplastic particles are wrapped in soil aggregates, which should be retained to prevent possible losses [29]. In addition, the heat deflection temperature (HDT) of PEs ranges from 40 °C to 82 °C. Therefore, the working temperature was controlled below 40 °C to protect the AMF-MPs in the soil from deformation.

### 2.2. Separation of MPs

The separation method in this study combined both density flotation and oil separation. We first tested three common solutions: ultrapure water (1.0 g/cm^3^, 18.2 MΩ·cm), sodium chloride (NaCl, 1.2 g/cm^3^), and sodium bromide (NaBr, 1.5 g/cm^3^) in containers to separate MPs by density differences, but there were still soil particles and impurities attached to the MPs in all three solutions, which caused some MPs to precipitate at the bottom of the container. NaCl was chosen as the solution due to its low cost, environmental friendliness, and moderate density. In addition, since ultrasonic cleaning was beneficial for removing foreign contaminants from the surface of MPs, we adopted the technology before each centrifugation to obtain MPs with adhered impurities. Ultrasonic cleaning technology can use high-intensity, high-frequency sound waves to generate cavitation bubbles in liquids. The energy released by the implosion of cavitation bubbles provides micro-stirring at the liquid-contaminant interface [36]. 

**Figure 1:**
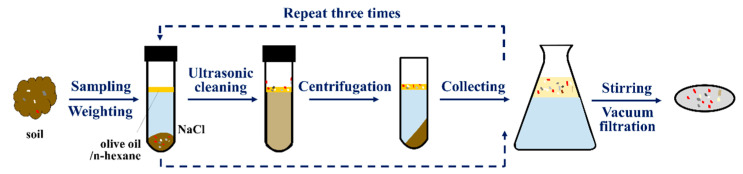
The schematic diagram of the separation method.

The separation method adopted in the current study includes four main steps: sample weighing, ultrasonic cleaning, centrifugation, stirring, and vacuum filtration (Figure 1).

A total of 10 g of each soil sample was weighed and added to a custom-made 100 mL polytetrafluoroethylene (PTFE) centrifuge tube. The tubes were then shaken for 0.5 min after adding 25 mL of saturated NaCl solution and placed in an ultrasonic cleaner (25 °C, 40 kHz) for 20 min. After adding 3 mL olive oil, the tube was shaken again and spun using a centrifuge for 10 min with the 2537 g RCF (relative centrifugal force). The resulting supernatant was then collected into a conical flask. The procedure was repeated three times, the last two ultrasonic cleanings were conducted for 10 min, and the solution used in the tube was n-hexane instead of olive oil during the third centrifugation.

In the third step, 30 mL of n-hexane solution was added to the conical flask, then placed on a magnetic stirrer, and stirred for 2 min at a speed of 1500 min^−1^. Before magnetic stirring, the mixed solution collected in the conical flask had three layers of stratification, n-hexane, olive oil, and saturated NaCl solution from top to bottom, due to density differences. After this step, the olive oil was fully dissolved in the n-hexane solution. 

Finally, the mixed solution was poured into a vacuum filtration unit and filtered through filter membranes allowing the separation of MPs to be accomplished.

In addition, MPs in soils can be separated in batches using the method. Olive oil was used as one of the separation solutions based on the mechanism of oleophilic interaction [18]. There is an attraction between the long-chain fatty acids of the oil and the polymer backbone, but the attraction is not strong enough to extract fluorinated polymers to the oil layer [17], so the material of the centrifuge tube is PTFE, which will not interfere with the experiment. After the first two centrifugations, a small amount of olive oil adhered to MPs remained in the centrifuge tube. It was necessary to dissolve the olive oil with n-hexane solution in the third centrifugation to collect MPs fully.

### 2.3. Digestion of Organic Matters

The separated MPs were still adulterated with organic matter, and we chose Fenton’s reagent as the digestion solution for the organic matter. The reagent’s composition includes 30% H_2_O_2_, 2 mmol/L FeSO_4_·7H_2_O, 2 mmol/L PCA (protocatechuic acid), and H_2_O (pure water), with a volume ratio of 10:1:1:5. The reduction ability of PCA could accelerate the Fe^2+^ cycle to enhance the digestion in the Fenton oxidation system [37]. The mixture obtained on the membrane after separation was rinsed into a conical flask using 30 mL of Fenton’s reagent. After covering the mouth of the flask with aluminum foil, they were kept in a constant temperature shaker overnight at 38 °C at a speed of 150 min^−1^. The amount of Fenton’s reagent and digestion time can be adjusted according to the performance of digestion. Then the MPs were collected by filtering and transferred to glass Petri dishes. At last, they were dried in an oven at 38 °C and stored for subsequent optical inspection.

### 2.4. MPs Identification and Quantification

All the obvious and possible AMF-MPs on filter membranes were identified using a stereo microscope (BX51, Olympus, Japan) at multiple magnifications of 50× and 100× and then were photographed with a digital camera equipped on the microscope. Undetermined MP-like particles were then confirmed by micro-Fourier Transformation Infrared Spectroscopy (μ-FTIR). Spectrum data were acquired using a μ-FTIR spectrometer (Nicolet iN 10 MX, Thermo Scientific, USA) at a resolution of 4 cm^−1^ with 24 scans for each spectrum in a spectral range from 400 to 4000 cm^−1^ [3,38]. The particle sizes ranged from 20 to 2000 μm. A sample μ-FTIR spectrum of AMF-MP is shown in Figure S2. Based on the μ-FTIR analysis, we confirmed the polymer types of the MPs by comparing their FTIR spectra with a reference database. Eventually, data of AMF-MPs, including shape, color, size, and quantity, were recorded with Image J software. The size was measured according to the longest dimension of MP particles [29]. The shape can be either fiber or film, and the color can be either transparent or black. More information on identification and experiments is described in detail in the supplementary material document.

In order to establish a model that can increase the efficiency of visual inspection, we performed repeated microscopic analyses of the confirmed AMF-MPs at different magnifications by using a stereo microscope and a scanning electron microscope (SEM) (Supra 55VP, Zeiss, Germany), and then summarized their characteristics. The results of the model are described in the next section.

### 2.5. Selection of Filter Membrane

**Figure 2:**
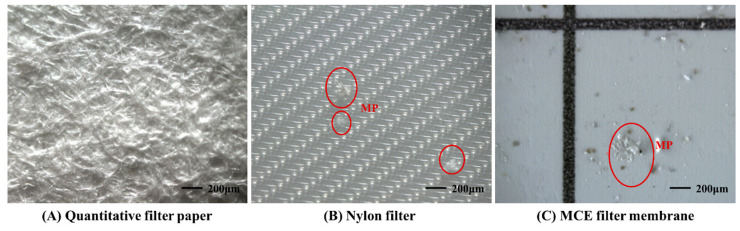
Microscopic photographs of various filter membranes: quantitative filter paper (**A**), nylon filter (**B**), and MCE filter membrane (**C**). Photograph (**B**) shows transparent MPs (red circle) hidden on the nylon filter.

During the experiment, MPs in solution were filtered several times by vacuum filtration. Glass fiber filter [3,17,38], quantitative filter paper [21,25], and nylon filter [39] are commonly used for MPs separation and organic matter digestion. We tested four types of filter membranes: glass fiber (pore size 1.6 µm), quantitative filter paper (maximum pore size 10–15 µm), nylon (pore size 20 µm), and mixed cellulose ester (MCE) (pore size 0.45 µm). It was found that the low toughness of the glass fiber filter made it difficult to resist breaking upon impact. Furthermore, previous research [39] proved that many fibers from glass fiber filters can be washed down with ultrapure water. Quantitative filter paper occasionally sacrificed integrity during vacuum filtration, mainly due to its weak strength and flexibility. Therefore, a nylon filter was often selected during the separation and digestion. However, none of the first three filter membranes were desirable when it came to identifying microplastics under a microscope. Subsequent analysis of the observation demonstrated that the brightness of the quantitative filter paper was too high and its surface texture was too messy to identify MPs (Figure 2A), and the translucent nylon filter hid transparent MPs (Figure 2B). In contrast, excellent color contrast was provided by the MCE filter membrane, which could better facilitate MPs identification and minimizes eye fatigue. In addition, the gridded surface (3.1 mm intervals) of the MCE membrane could assist in manual MP counting (Figure 2C). Consequently, the MCE filter membrane was selected for the last filtration before identifying microplastics in the current study, and the nylon filter membrane was selected for the other filtrations.

The details of the main apparatuses and consumable materials used in the current study are listed in Table S1.

### 2.6. Controlled Experiments and Evaluation

The optimization of methods is described in Section 3. In order to evaluate the effect of optimization, we conducted controlled experiments using real soil samples. The samples were divided into two groups, the experimental group treated with the optimized method and the control group treated with the original method. The soil samples used for method evaluation were taken in the same farmland as the studied samples and were heated (550 °C overnight) to remove the original MPs and all types of organic matter [35]. In order to simulate possible AMF-MPs in farmland soils, we used different types of AMFs to make experimental MPs through multiple shearing and grinding. These AMFs differed in material type (LDPE, LLDPE, and HDPE), thickness (8–14 μm), and color (transparent and black). Each soil sample was randomly spiked with 1.0–2.0 mg self-made MPs [16,25]. More details are described in the supplementary material document. We separated and collected the MPs in the simulated soil samples using successive methods described earlier. Upon collection with filter membranes, they were dried to a constant weight at 38 °C in an oven, and then the weight of separated MPs was obtained by the difference before and after ignition (550°C, overnight). To ensure the accuracy of evaluation, each soil sample was evaluated in triplicate, and quantitative filter paper (maximum pore size 10–15 µm) was used in the last filtration because of its ashless feature. The recovery ratio of MPs can be calculated based on the formula shown below:

R = W_1_/W_2_ ×100% (1)

In Equation (1), W_1_ is the weight of separated MPs, and W_2_ is the weight of added MPs.

To avoid contamination during the experiment, the vessels for research were glassware or metalwork, which were rinsed with ultrapure water before use, and materials that could release MPs were discarded, including clothing and sampling tools [39]. In order to prevent MPs contamination from the atmosphere, the vessels without lids were covered by aluminum foil as much as possible. Especially, blank samples (ultrapure water without MPs) were set up throughout the process to identify ambient contamination [40].

## 3. Results and Discussion

### 3.1. Optimization of the Methods

#### 3.1.1. Accelerating Filtration

Existing methods describe the operation of vacuum filtration very briefly; however, due to a small amount of impurities in the supernatant or other reasons, filtration in real applications is usually time-consuming and prone to clogging. These limitations also appeared in the filtration of the mixed solution in our approach. In addition, when the mixed solution was stirred, a small amount of emulsion would be produced. Furthermore, stirring at high speeds brought air into the solution and resulted in bubbles, which would clog filter membranes and hinder filtration. 

**Figure 3:**
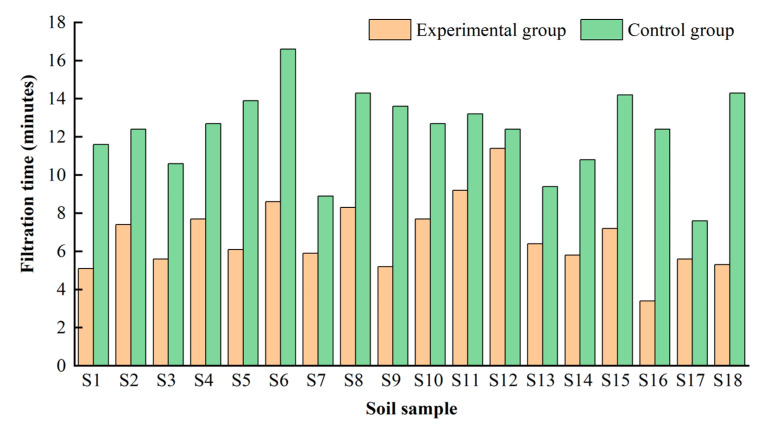
Control experiment on filtration time for different soil samples.

In order to promote filtration, we left the mixed solution to stand for 1 h to remove bubbles, then a larger volume of ultrapure water (200–500 mL) and the mixed solution were poured into the funnel at the same time to dilute the emulsion and rinse filter membranes. The time required to filter the mixed solution with the above treatment was tested using controlled experiments, where the measure was used in the experimental group but not in the control group. As shown in Figure 3, filtration times for the eighteen soil samples in the control group ranged from 7.6 to 16.6 min, with an average of 12.3 min. Filtration times of the experimental group ranged from 3.4 to 11.4 min, and the average time was 6.8 min, which was shortened by nearly half. 

Consequently, the limitation of the filtration speed was greatly overcome. The results confirmed that rinsing filter membranes with a large amount of ultrapure water can avoid blockage.

#### 3.1.2. Purification of MPs

After the digestion of organic matter, a few extremely tiny grits from the soil still remained and affected the purity of the MPs [35], making it necessary to perform purification. The MPs were rinsed into a conical flask again with saturated NaCl solution and were left to settle. Finally, the resulting supernatant was filtered to collect the MPs. 

**Figure 4:**
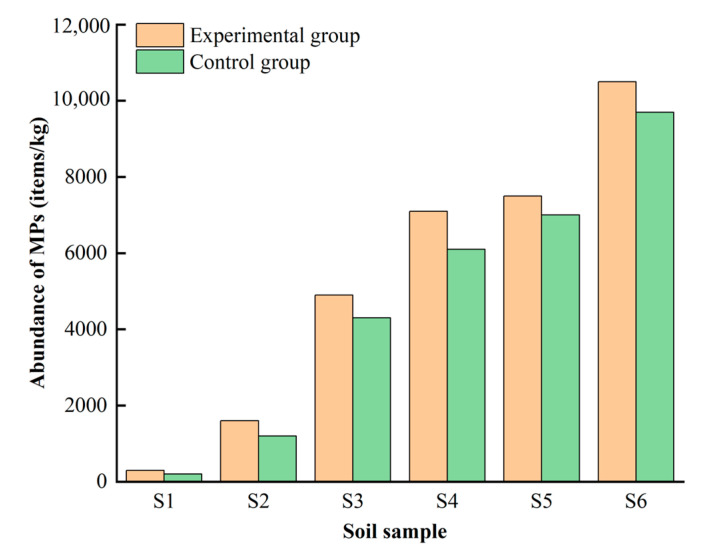
Control experiment on the abundance of MPs identified in different samples.

As shown in Figure 4, six real soil samples with different mulching film ages were used to conduct control experiments to demonstrate the impact of purification on MPs identification. MPs in the experimental group were purified after digestion, while those in the control group were not. The mulching film ages of samples, shapes, colors, and sizes of MPs were identified in different samples and are listed in Table 1.

**Figure 5:**
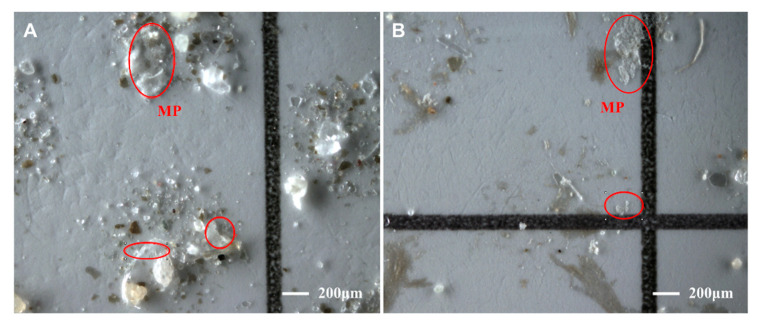
Comparison of microscopic analysis of MPs without (**A**) and with (**B**) purification. The red circles represent MPs, and the MPs in image (**B**) are not interfered with by impurities and are easier to identify than those in image (**A**).

Generally, an older mulching film age leads to more MPs being present. The abundance of MPs in sample S6, which was taken from a field with a mulching film age of 34 years, was 10,500 items/kg, the largest in all samples studied (Figure 4). The properties of the MP particles identified in the two groups of samples were the same in terms of shape and color. However, generally, there are more MP items identified in the experimental group than in the control group, and the MPs in the experimental group were generally smaller in size. It is shown that some small-sized (57–141 μm) particles are missing for unpurified MPs after digestion (Table 1). Through microscopic analysis of the samples, it can be found that there are plenty of extremely tiny grits and other impurities on filter membranes in the control group (Figure 5A). Therefore, impurities hid MPs and hindered identification, which would take more time to observe. The experimental group was cleaner due to fewer impurities, and the MPs on them were clearer and more obvious (Figure 5B). The control experiment revealed that the purification of MPs can accelerate identification. In addition, this experimentally validated approach can contribute to a more accurate accounting of the number of MPs in soils.

### 3.2. Recovery of MPs

**Figure 6:**
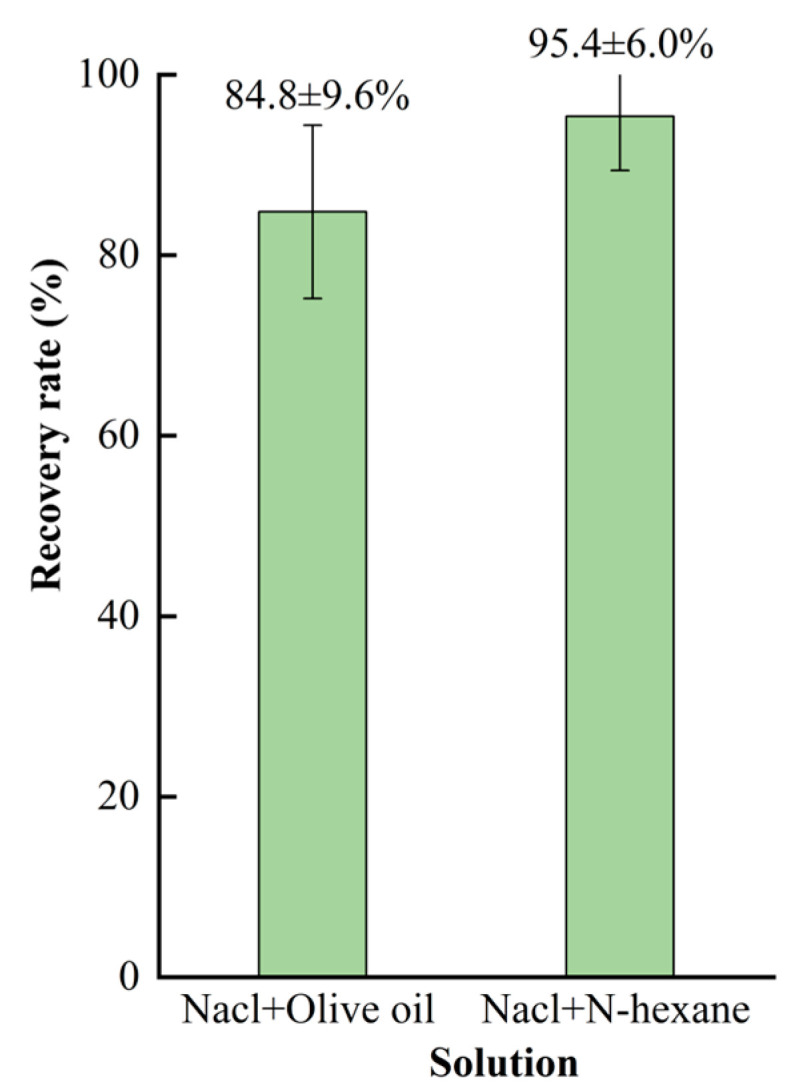
Recovery rate (mean ± SE) of MPs in soil using different solutions during the third centrifugation.

The existing methods often use the same solution when repeating the MP separation experiment. In this study, after three repeated centrifugations using a mixed solution of saturated NaCl solution and olive oil, we found that a little olive oil was still attached to the inner wall of the centrifuge tube, which caused some remaining MPs to be retained in the tube. More MPs could be obtained when n-hexane was used instead of olive oil during the third centrifugation. We further evaluated the MP recovery rate with the separation method using different solutions during the third centrifugation (Figure 6). The mean recovery rate is 84.8 ± 9.6% (n = 18) with a mixed solution of NaCl and olive oil and 95.4 ± 6.0% (n = 18) when the mixed solution was NaCl and n-hexane. The purpose of using n-hexane is to dissolve the residual olive oil and enhance its fluidity so as to obtain more microplastics, which is based on the principle of “like dissolves like.” The results demonstrate that adding n-hexane to tubes during the third centrifugation can improve the recovery rate of MPs.

### 3.3. An AMF-MPs Identification Model

**Figure 7:**
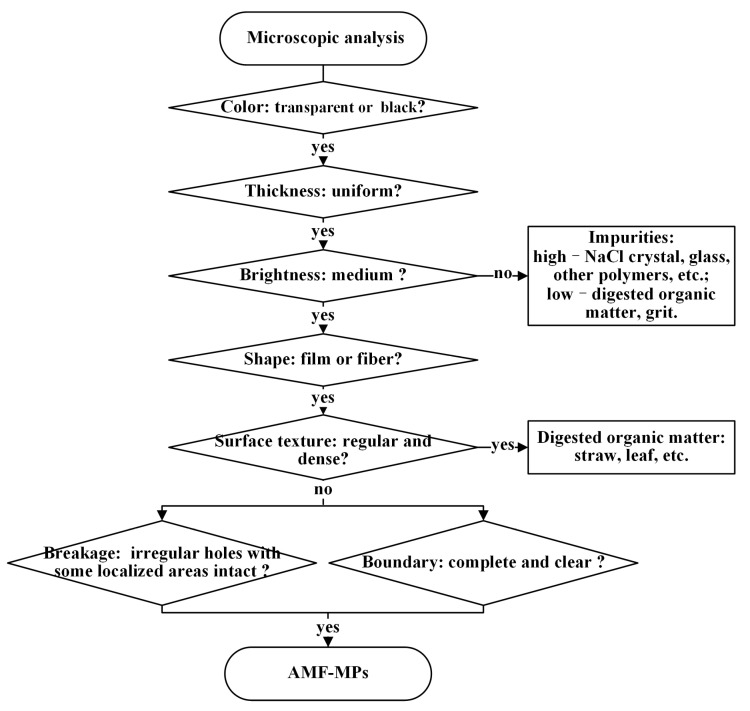
AMF-MPs identification model.

We proposed an AMF-MPs identification model (Figure 7) based on the following characteristics:

(a) Color: transparent or black. Most of the agricultural mulching films used in the study area are transparent, and a few are black, which determines the color of the MPs. Impurities with other colors can thus be excluded.
(b) Thickness: uniform. The fibrous MP curls up and becomes thinner at both ends (Figure S3).
(c) Brightness: medium. As shown in Figure S4, MPs originating from transparent films often exhibit lower brightness than NaCl crystals, glass, and other polymer fragments, mainly due to their lower reflectivity. It becomes brighter only when light is reflected from the folds. In addition, the brightness of MPs originating from transparent films is higher than that of the film-like digested organic matter.
(d) Shape: film or fiber.
(e) Surface texture: irregular. Contrary to AMF-MPs, the surface of digested organic matter such as straw and leaves will have regular and dense textures.
(f) Breakage: irregular holes with some localized areas intact. The size and distribution of holes are both irregular. In contrast to organic matter, MPs do not have penetrating cracks due to their good flexibility.
(g) Boundary: complete and clear. MPs have white and clear outlines due to refracted light.

**Figure 8:**
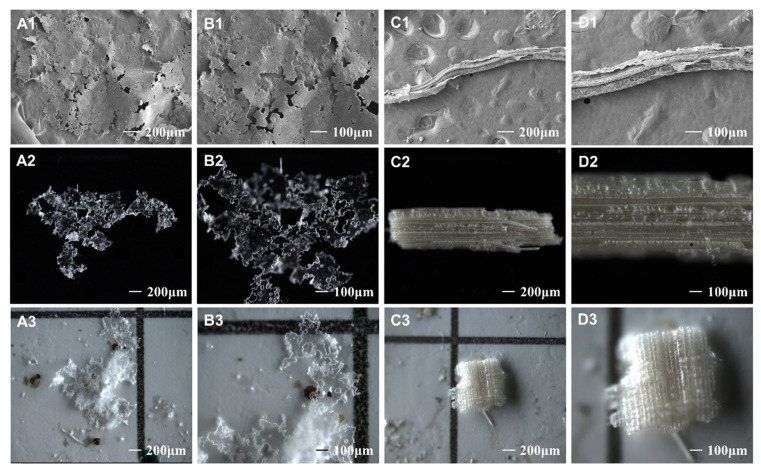
Observation of MPs and organic matters under stereoscopy: (**A1**–**A3**,**B1**–**B3**) transparent AMF-MPs separated from soils; (**C1**–**C3**,**D1**–**D3**) digested organic matters; multiple magnifications of (**A1**–**A3**,**C1**–**C3**) 50× and (**B1**–**B3**,**D1**–**D3**) 100×. (The first line shows SEM images; the last two lines, respectively, show microscopic images with a background of black and MCE filter membrane.).

Figure 8 shows the characteristics of MPs and digested organic matter at multiple magnifications. After eliminating obvious impurities during the visual inspection, MPs can be identified using the AMF-MPs identification model. The most typical characteristic of AMF-MPs is uniform thickness, which is due to the feature of AMFs. The second significant characteristic is surface texture. The surfaces of digested organic matter, such as straw and leaves, often show regular and dense grids or lines, which are the original structures of plants. However, MPs have been in the soil for a long time, resulting in the formation of tiny holes with irregular shapes and sizes due to wear, corrosion, and decomposition. Additionally, when MPs are small enough, some of their characteristics may be lost. Our batch experiments using the AMF-MPs identification model found that the minimum size of MPs identified was 51 ± 3 μm.

### 3.4. Time and Economic Cost

**Figure 9:**
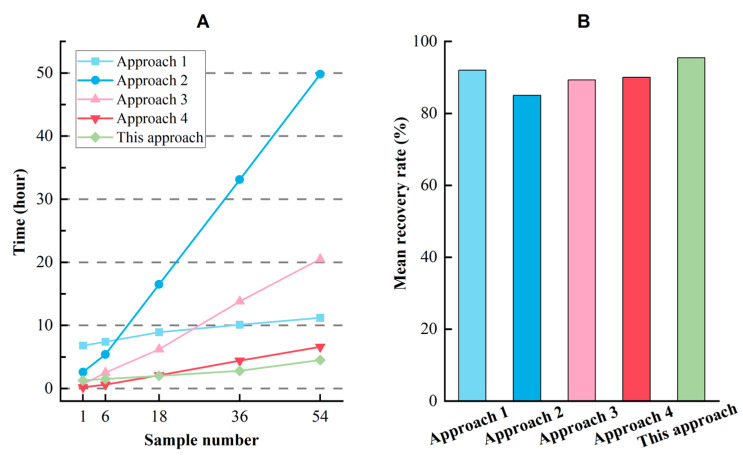
(**A**,**B**) Time cost and mean recovery rate of the previous and this approach. Approach 1 to Approach 4 were from references of [17,20,21,25], respectively.

To determine the time and economic cost of implementing this approach, we conducted batch experiments using actual soil samples. The efficiency of the batch experiments was found to be higher when more advanced experimental apparatuses were used. We utilized three sets of common laboratory apparatuses (listed in Table S1) for the separation experiments, allowing us to process up to 18 soil samples per batch. Multiple batches of experiments can be conducted consecutively. For instance, as illustrated in Figure 9, the separation of MPs from 54 soil samples can be completed in 4.5 h using this approach.

We conducted a comparison of our approach with four existing methods, assuming the same number of apparatuses were used based on separation times described in previous studies [18,21,22,26]. Our findings show that as the sample number processed in batches increased, the separation time for MPs decreased using our approach (Figure 9A). Additionally, our approach yielded a higher mean recovery rate for MPs made of PE material compared to the other methods (Figure 9B). 

Moreover, our approach allowed for the synchronous processing of soil samples, digestion, and purification of each batch, without any time conflicts. As a result, the time needed for these two steps can be ignored. We also utilized the AMF-MPs identification model to identify and quantify MPs, and each sample took an average of 20 to 30 min to process. The purchase cost of consumable materials and reagents for each soil sample using our method was approximately 2.5 US dollars.

Our data indicate that conducting batch experiments using the proposed approach is more efficient in terms of time and cost compared to other approaches.

## 4. Conclusions

Accurately and rapidly separating and identifying microplastics in soil remains a big challenge, especially when a large number of soil samples need to be analyzed. We proposed a method for separating microplastics, which combines density separation and oil separation, whereby a solution of olive oil (or n-hexane) mixed with saturated sodium chloride is utilized. The average recovery rate of MPs was found to be 95%, which was confirmed through control experiments that demonstrated how rinsing filter membranes with ultrapure water could increase the filtration rate and purification after digestion could yield more accurate data on MPs in soils. Using a model, we were able to identify the minimum size of MPs as 51 ± 3 μm. This method was able to separate MPs from 54 soil samples in just 4.5 h, with a cost of approximately 2.5 USD per soil sample. Compared with previous methods, this approach had a shorter separation time and a higher mean recovery rate, making it easier to implement in batches due to the availability of common laboratory equipment and consumable materials. Given olive oil’s strong affinity with most polymers, further studies are needed to test this approach with MPs of different materials and densities to determine its applicability in diverse soil environments.

## Figures and Tables

**Table 1:** The sample mulching film ages, shapes, colors, abundance, and size ranges of AMF-MPs identified in different samples with purification (experimental group) and without purification (control group).

Soil Sample	Mulching Film Age (Year)	Experimental Group				Control Group			
		Shape	Color	Abundance of MPs (Items/kg)	Size Range (μm)	Shape	Color	Abundance of MPs (Items/kg)	Size Range (μm)
S1	3	Film	Black, Transparent	300	101–2809	Film	Black, Transparent	200	1235–2809
S2	7	Film, Fiber	Transparent	1600	142–3624	Film, Fiber	Transparent	1200	445–3624
S3	16	Film	Transparent	4900	113–4088	Film	Transparent	4300	206–4088
S4	20	Film, Fiber	Black, Transparent	7100	94–4105	Film, Fiber	Black, Transparent	6100	135–4105
S5	28	Film, Fiber	Transparent	7500	57–4187	Film, Fiber	Transparent	7000	172–4187
S6	34	Film, Fiber	Transparent	10500	73–3961	Film, Fiber	Transparent	9700	121–3961

## Data Availability

No data are available at the moment due to privacy requirements.

## Supplementary Materials

The following supporting information can be downloaded at: https://www.mdpi.com/article/10.3390/toxics11050461/s1. More detailed descriptions of microscopic analysis; Figure S1: Location of sampling sites; Figure S2: The μ-FTIR spectrum of AMF-MP: the polymer type is PE; Figure S3: Observation of MPs under stereoscopy: (A) black film MPs; fiber MPs in different colors of (B) black and (C) transparent; Figure S4: Distinguish between MPs (red circle), organic matters (orange circle) and impurities (blue circle) on MCE filter membranes. Images showing examples of MPs in different colors and shapes; Figure S5: Microscope used to identify MPs: Equipping with white side light can enhance the three-dimensional effect of collections; Table S1 Main apparatus and consumable materials of this study [41,42,43,44].
