# Supplementary material for: A Low-Cost Approach for Batch Separation, Identification and Quantification of Microplastics in Agriculture Soil
Source: Toxics. 2023 May 16;11(5):461. doi: 10.3390/toxics11050461 (PMC10221660; doi:10.3390/toxics11050461)
Supplement: Supplementary file 1 [file toxics-11-00461-s001.zip › toxics-2266141-supplementary.pdf]

Supplementary materials

Materials and Methods

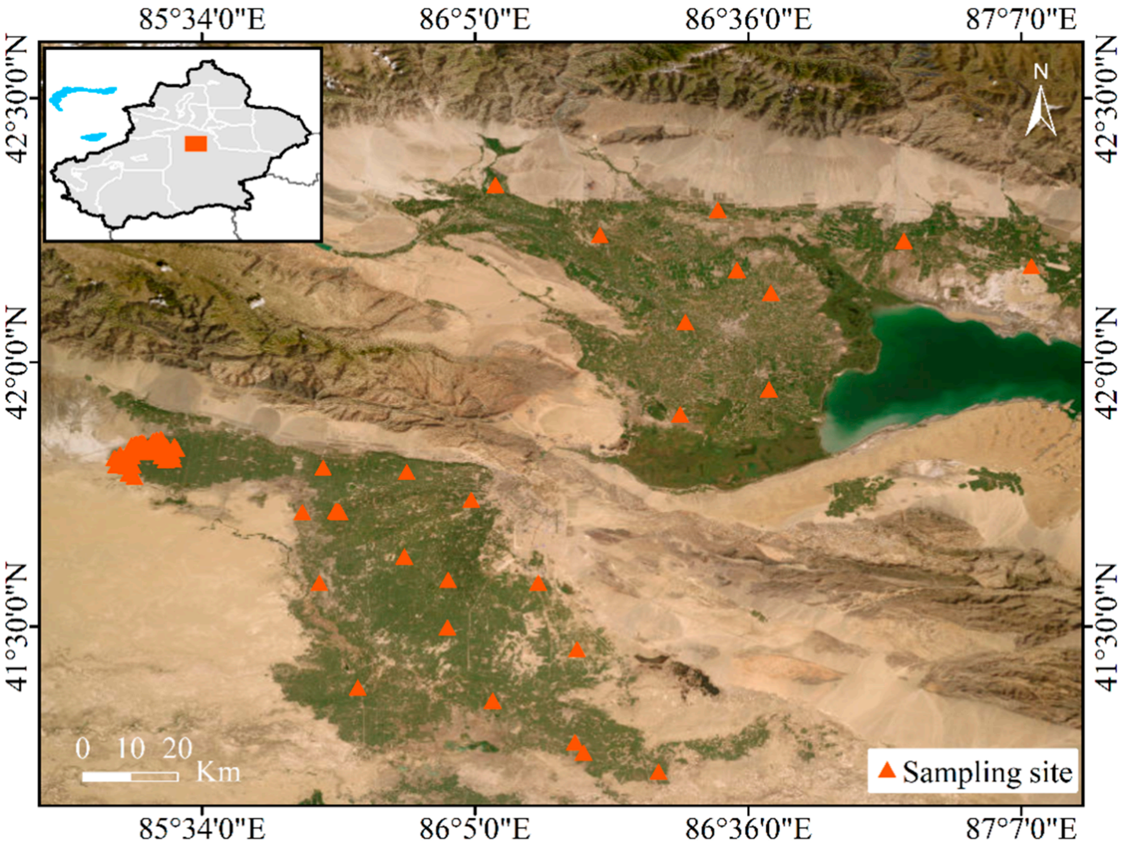

Fig S1. Location of sampling sites.

Table S1 provides a detailed list of the main apparatuses and consumable materials used in our study. In order to simulate potential agricultural soil contamination by AMF-MPs, we created test microplastics using various types of films with different material compositions (LDPE, LLDPE, and HDPE), thicknesses (ranging from 8–14 μm), and colors (transparent and black). The fabrication process for these test microplastics involved [16,25]: (1) shearing and grinding the AMFs multiple times to create MP particles, (2) sorting the MP particles into four size categories: <50 μm, 50–100 μm, 100–250 μm, and >250 μm, and (3) thoroughly mixing the four categories of MPs in weight ratios of 0.21%, 1.49%, 38.13%, and 58.35%. In the pre-experiment, we collected some soil samples from croplands without mulching history and detected AMF-MPs. In addition, Zhang et al. [41] demonstrated that microplastics were detected in the atmosphere of urban, suburban, and even remote areas far from source regions of microplastics, indicating that the potential long-distance atmospheric transport for microplastics. Therefore, this study heat-treated (550℃, overnight) the soil samples to make them clean and free of microplastics [4]. Finally, 1.0–2.0 mg of the resulting test microplastics were randomly added to each clean soil sample. In previous microplastic extraction methods [32,42-44], organic matters in soils were digested before microplastic separation, so it is reasonable to add microplastics to the soil without organic matter when we evaluate the recovery rate of microplastics.

Table S1. Main apparatus and consumable materials of this study

Item	Manufacturer	Tradename	Location	Specification & Description
Centrifuge	Thermo Scientific	X1R	USA	/
Nylon filter	Millipore	NY2004700	USA	Pore Size: 20μm, diameter :47mm
Mixed cellulose ester filter membrane	Whatman	7141-104	UK	Pore Size: 0.45μm, diameter :47mm
Quantitative filter paper	Newstar	NS203	China	Ash percent≤0.01%, diameter :70mm
Stereo microscope	Olympus	BX51	Japan	/
Micro Fourier-transform infrared spectroscopy spectrometer	Thermo Scientific	Nicolet iN 10 MX	USA	/
Scanning electron microscope	Zeiss	Supra 55VP	Germany	/

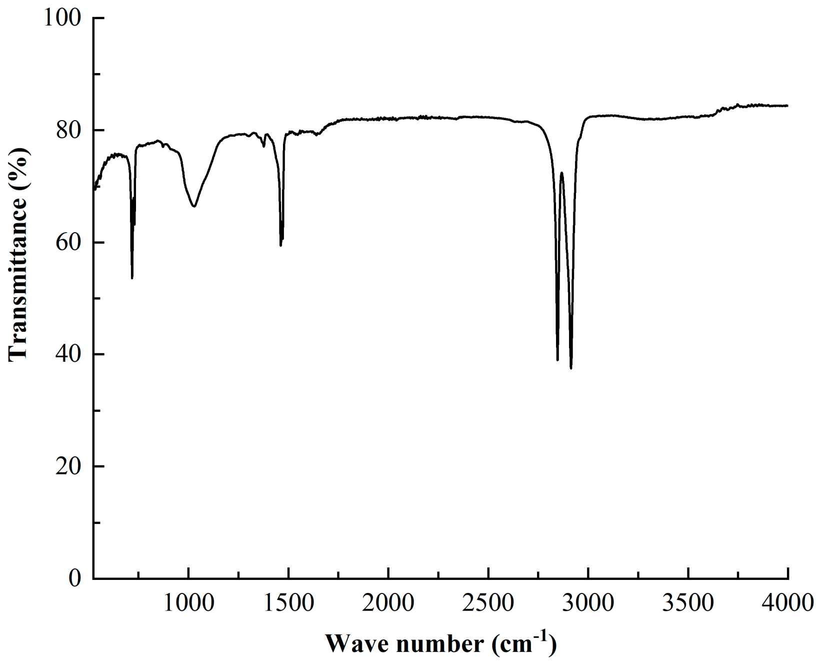

Figure S2. The μ-FTIR spectrum of AMF-MP: the polymer type is PE.

Results and discussion

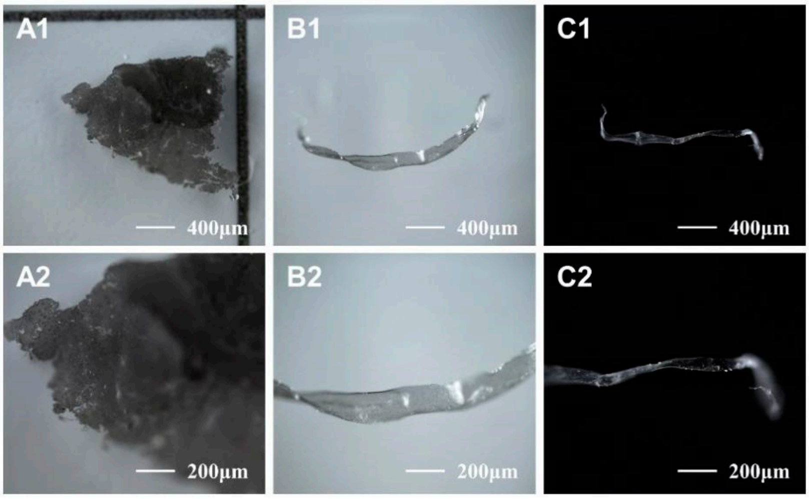

Fig S3. Observation of MPs under stereoscopy: **(A)**black film MPs; fiber MPs in different colors of **(B)** black and **(C)** transparent.

Fig S3 illustrates the observation of different types of MPs under stereoscopy. Panel A shows black film MPs, while panels B and C display fiber MPs in black and transparent colors, respectively.

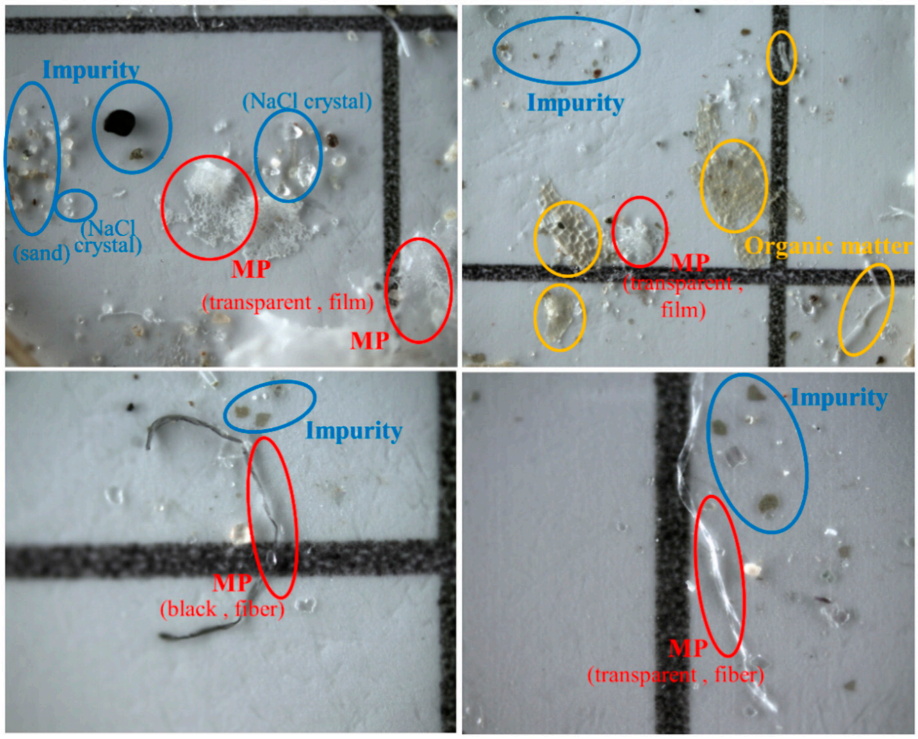

Fig S4. Differentiate among MPs (red circle), organic matters (orange circle), and impurities (blue circle) on MCE filter membranes. The images depict various examples of MPs, each with distinct colors and shapes.

Figure S4 presents several examples of how to distinguish MPs from organic matter and other impurities on MCE filter membranes.

To aid in microscopic analysis, we utilized a white side light with a color temperature of 4500 K to improve the three-dimensional appearance of the collections on the MCE filter membrane and simplify visual inspection, as demonstrated in Fig S5.

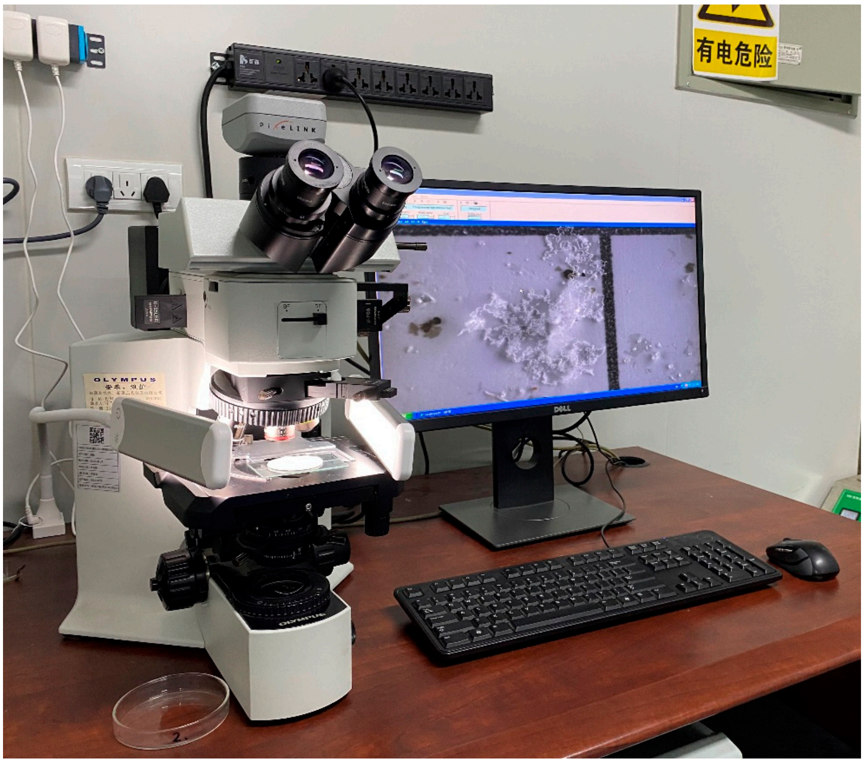

Fig S5. The microscope used to identify MPs, equipped with a white side light to enhance three-dimensional effect of collections.
